# Oxygen Gas

**Published:** 1876-10

**Authors:** G. B. Heard

**Affiliations:** LaGrange, Ga.


### OXYGEN GAS.

By G. B. HEARD, M.D,. LaGrange, Ga.

Case VIII.—On 17th day of last February, M. A. Turner, of this city, called upon me on account of lung trouble, stating that during the war he was discharged from service by an army medical board, on account of consumption. Has had several hemorrhages. Auscultation revealed a consolidated condition of the left lung, muco-purulent expectoration, and severe pains through the chest. I gave him daily, morning and evening, four gallons of oxygen. It is to be observed that his health, previous to last winter, had been comparatively good. His cough was severe, and night sweats abundant; no appetite. There was a gradual improvement in his condition, and on the 15th of March he was discharged as being well, and to-day appears to be in perfect health.

Case IX.—Mrs. B., of this place, has been feeble for nearly two years. Has been confined to the house since December last. Dr. Baugh, her attending physician, has kindly furnished me the notes in this case, having attended her occasionally up to January, and after that time gave her daily attention. She was extremely emaciated, with distressing cough, and purulent expectoration, presenting all the rational signs of incipient pulmonary consumption. Anorexia was absolute, night sweats profuse, pulse ranging from 115 to 130 per minute. The usual treatment was had in her case, without any amelioration of the symptoms.

On 17th of February, at my suggestion, he gave her four gallons of oxygen in a free admixture of common air, occupying about one hour in inhaling it. In a few days the area of dulness began to lessen, cough and expectoration to diminish, appetite to improve, and she gradually returned to perfect health, and was discharged after six weeks treatment, as being well.

Case X.—T. R. Gould, Stanford, Conn., age 27; phthisis. Has been sick five years. Began treatment April 1st, 1876. Weight 119 pounds; very pale and anæmic; distressing cough and profuse expectoration; exhausting night sweats; constant fever; pulse ranging from 96 to 120 per minute; respiration

35; anorexia, complete tubular breathing at apex of left lung, with feeble respiratory murmurs in the lower portion. Gave him four gallons of oxygen in the morning. It had a very exhilerating effect; slept better than usual that night.

*April 2d.* Four gallons. Condition seemed to be about the same as yesterday.

*April 3d.* Same amount given. Appetite much improved; slept well; respiration easier; circulation 90.

*April 5th.* Cough less, and the expectoration not quite as profuse; circulation 84; respiration 20; able to take a longer walk than before for months, without much fatigue. Continued same treatment.

*April 8th.* Fever entirely gone; no night sweats; ravenous appetite; cough greatly lessened, and expectoration very much diminished. The patient weighed to-day 121½ pounds.

*April 10th.* Coughs but little; sleeps well, and says that he feels as well as ever he did. Took a long walk without any fatigue.

*April 12th.* He says that he feels all right, and desires to be absent from treatment a few weeks.

*June 5th.* Patient returned, and the change in his appearance is wonderful; he is full in the face, with a healthful appearance, and walks about with an elastic step not known to him for years. His weight is to-day 134½ pounds avoirdupoise.

*August 16th.* I made a critical examination to-day, and find nothing abnormal in respiratory organs.

I have been particular in reporting this case, from the fact that the patient had no other treatment than the oxygen.

Case XI. Dr. Murphy, of this place, had suffered for years with asthma. In the spring of 1875, he began the oxygen inhalations, and kept the treatment up about six weeks. In a conversation with him a short time ago, he said nothing could be more satisfactory than the effects of it on him, there being no return of the attack in eighteen months.

Case XII.—Mr. J. T. Jourdan, of Midway, Alabama, has had asthma for four years, with great flatulence. Began treatment August 10th. The flatulence in the stomach disappeared in three or four days, and now sleeps well, without any paroxysm. Says that he feels as well as he ever did, yet it is thought best to continue the treatment a few days longer. The only additional treatment to the oxygen is the following:

℞.—Iodide Potassium, . . . . . ʒiiss.

Aqua Cinnam., . . . . . . ℥iv.

S. Take teaspoonful three times a day.

